# Application of Waste Glycerol as a Draw Solution for Forward Osmosis

**DOI:** 10.3390/membranes12010044

**Published:** 2021-12-29

**Authors:** Ewa Bernacka, Hanna Jaroszek, Marian Turek, Piotr Dydo, Dymitr Czechowicz, Krzysztof Mitko

**Affiliations:** 1 PolymemTech Sp. z o.o., Ul. Wołodyjowskiego 46, 02-724 Warsaw, Poland; h.jaroszek@gmail.com; 2 Faculty of Chemistry, Silesian University of Technology, Ul. B. Krzywoustego 6, 44-100 Gliwice, Poland; marian.turek@polsl.pl (M.T.); piotr.dydo@polsl.pl (P.D.); dymitr.czechowicz@polsl.pl (D.C.); krzysztof.mitko@polsl.pl (K.M.)

**Keywords:** forward osmosis, waste glycerol, draw solution

## Abstract

Waste glycerol generated during biofuel production accounts for ~10% of total biodiesel volume. Increasing the use of renewable energy sources, including so-called biodiesel, will significantly increase the amount of waste glycerol for disposal. One possible route for waste glycerol reuse is to use it as a draw solution in forward osmosis (FO). Glycerol solutions are particularly suited as FO draw solutions due to their high osmotic pressures. In this work, the effects of waste glycerol composition on FO draw solution osmotic pressures, as well as the effects of membrane type and linear flow velocities on FO water and reverse flux, were investigated. Those results indicated the feasibility of using waste glycerol as a draw solution in FO, allowing the reuse of significant amounts of this by-product.

## 1. Introduction

Usage of biofuels—fuels produced from biomass—is increasing worldwide; however, their production often generates many unwanted by-products. Several methods to produce biofuels from vegetable oil have been developed: thermal and catalytic cracking, pyrolysis, microemulgation, and, the most common method, transesterification [1]. Transesterification produces esters (biodiesel) and glycerol, the waste by-product. Glycerol accounts for approximately 10% of the total biodiesel production volume [2,3] and usually contains 50–85% glycerol, inorganic salts (NaCl, KCl, or K_2_SO_4_, depending on the production technology), and methanol [4]. The actual product composition depends on the quality of the compounds used, the molar ratio of oil to methanol, the type and quantity of catalyst, the process temperature, stirring speed, separation time, and pressure. Increasing demand and production of biofuels requires utilizing a large amount of waste glycerol, and there are several methods for doing so. Unpurified waste glycerol may be used as a supplemental energy source for cattle, broiler, and pig food, or to dilute liquid fertilizers [5]. Due to its high energy content, crude glycerol is used directly as boiler fuel or for the co-generation of electricity and thermal energy. The largest glycerol consumers are, however, the pharmaceutical, chemical, and cosmetic industries, which require purification and concentration of waste glycerol prior to use. Proper treatment technologies are selected based on customer needs [1]. Industrial purification of crude glycerol usually requires the removal of organic pollutants, desalination (which usually requires dilution of the dense crude solution), and dehydration. As its thermal instability makes simple distillation ineffective, ion exchange and vacuum distillation are two commonly used methods. This has led to the rising need for inexpensive methods of purification and concentration of waste glycerol.

Forward osmosis (FO) is a relatively new membrane process and requires less energy than other dehydration methods [6,7]. It is based on the transport of water through a dense semi-permeable membrane, similar to reverse osmosis (RO); however, unlike RO, FO does not require high pressures. The driving force of forward osmosis is the osmotic pressure difference across the membrane. Water is transported from the feed solution—a solution of low osmotic pressure (e.g., freshwater, river water) into the so-called draw solution—a solution of high osmotic pressure (e.g., sea water, waste glycerol)—via osmosis. Solutes influencing the osmotic pressure of the solution (e.g., salt, ionic liquids, organic compounds) are transported into the feed solution (low concentration) due to the difference of chemical potential on both sides of the membrane [8]. Forward osmosis operates at lower hydraulic pressures than common pressure-driven methods: for instance, the pressure range for reverse osmosis is 5–70 bar; for nanofiltration, the pressure ranges from 5–40 bar. However, for forward osmosis, the required pressure ranges from 0–5 bar. Forward osmosis is environmentally friendly and follows green chemistry principles. Forward osmosis has applications in water desalination, brine concentration, water softening, wastewater purification, and production of drinking water from seawater [9,10,11]. Various aspects of forward osmosis, such as the draw solution [12,13], the membranes used [14,15,16,17,18], fouling and scaling [19,20], flux [21], and energy consumption [7], have been reported previously. The most commonly used draw solutions are inorganic salts, such as MgCl_2_, NH_4_HCO_3_, and Al_2_(SO_4_)_3_, or organic compounds, such as glucose, fructose, and methanol, or organic salts [13,22].

High osmotic pressure makes glycerol solutions suitable draw solutions for forward osmosis [21,23]. For example, waste glycerol was applied in laboratory experiments in a crude glycerol fermentation biorefinery as a draw solution for dehydration of butanol obtained during crude glycerol fermentation [24,25]. In addition, FO can be used as an energy-saving alternative for glycerol dehydration, as compared to more commonly used vacuum evaporation. This study aims to prove that crude glycerol can serve as an FO draw solution for partial dewatering of purified glycerol from biodiesel production to obtain a >95% glycerol solution, pure enough for pharmaceutical/cosmetological applications. The proposal is to effect the simultaneous concentration of purified glycerol while using the water removed to dilute the crude glycerol.

The water flux in FO, thus the dehydration rate and achievable concentration, depends on its driving force—osmotic pressure difference—while so-called reverse fluxes of compounds contained in the draw solution (impurities transported from draw solution to feed solution) are diffusion-driven and affected by concentration differences and flow conditions. To maximize FO efficiency, the highest possible osmotic pressure gradient and the lowest possible reverse flux of impurities should be maintained during the process. To achieve this: (a) the effects of processing parameters, such as the membrane and linear flow velocity, should be determined; (b) a draw solution with the highest possible osmotic pressure should be selected; and (c) the water flux dependence on the driving force should be determined. This work addresses these aspects and formulates criteria to assess waste glycerol as a forward osmosis draw solution with the potential for additional applications in designing crude glycerol purification processes that produce high purity glycerol.

## 2. Materials and Methods

### 2.1. Model Solutions of Waste Glycerol

Model solutions of waste glycerol were prepared by mixing pharmaceutical-grade glycerol (Bio-Chem Sp. z o.o.) and model impurities: inorganic salts: NaCl, purity (99.5%, POCH); organic impurities: methanol, pure (99.8%, POCH). Ultrapure water with a resistivity of 15 MΩ·cm was produced with an Elix Essential Water Purification System (Merck Millipore, Burlington, MA, USA) throughout the experiments. Waste glycerol often contains ash but was not considered in this study because it does not transport through membranes.

### 2.2. Analysis of Impurity Levels

Concentrations of ionic impurities were determined by ion chromatography (Ion Chromatograph Dionex ICS-5000+ Reagent-Free HPIC System). Methanol and glycerol concentrations were determined by gas chromatography (Shimadzu GC-2010, equipped with an FID detector, autosampler, stove with programmable temperature ranging from 40–450 °C).

### 2.3. Osmotic Pressure Determination

As the difference of osmotic pressure between feed and draw solution drives the FO process, the osmotic pressure of each solution was the most important parameter to achieve the high water flux. Osmotic pressure, as well as freezing point depression, is a colligative property and derives from Raoult’s law. Osmotic pressure was calculated based on freezing point by a combination of equations for osmotic pressure and freezing point [26]. Freezing points of the glycerol solutions were determined using an osmometer 800CLG (Trident Med, Warszawa, Poland) with an additional Peltier cell, which allows the cooling temperature to lie in a range from −7 to −38 °C (thus allowing measurement of osmotic pressure from 0–400 bar with a measurement uncertainty of ±15%). Values exceeding the measurement range were extrapolated using Statistica 13.1 software.

### 2.4. Diffusion Experiments

Water flux and reverse flux of solutes are the two most important parameters characterizing transport through a membrane, and thus FO efficiency. They depend on the driving force and the membrane properties.

The first part of the FO experiments measured water and reverse fluxes through the membrane to select the best FO membrane. These measurements were conducted in a batch mode in a self-constructed laboratory module consisting of two chambers with a magnetic stirrer (1200 rpm) in each chamber to minimalize the concentration polarization effect (the effect of stirring rate was not verified on concentration polarization), separated by the semi-permeable flat-sheet membrane under investigation. The effective membrane area was 60 cm^2^. The feed was 500 mL of ultrapure water, whereas the draw solution was 350 mL of either 40% pure glycerol, or 40% glycerol with contaminants (3% NaCl and 5% methanol). All experiments were conducted at 21 °C. Aliquots (0.5 mL) were taken every 15, 30, or 60 min depending on the water flux.

Three types of membranes were examined (see Table 1). RO and NF membranes were investigated to determine whether it was possible to restrain the reverse flux of impurities relative to the FO membrane. RO and NF membranes are characterized by a thick support layer (unlike FO membranes) to withstand high pressure, which could become a severe limitation when used as an FO membrane because the internal concentration polarization (ICP) diminishes the water flux. Membranes were rinsed and soaked in clean ultrapure water for at least 24 h for conditioning and removal of the preservatives before the experiments were conducted.

After membrane selection with the highest water flux and relatively low reverse flux, the diffusion of methanol, salt, and glycerol from draw solutions (comprising 20–80% glycerol, 5% methanol, 1–5% NaCl—the data range of variability in the composition of waste glycerol in the literature [2]) through the selected membranes was investigated. All experiments were conducted in batch mode under the same conditions as during membrane selection.

**Figure 1:**
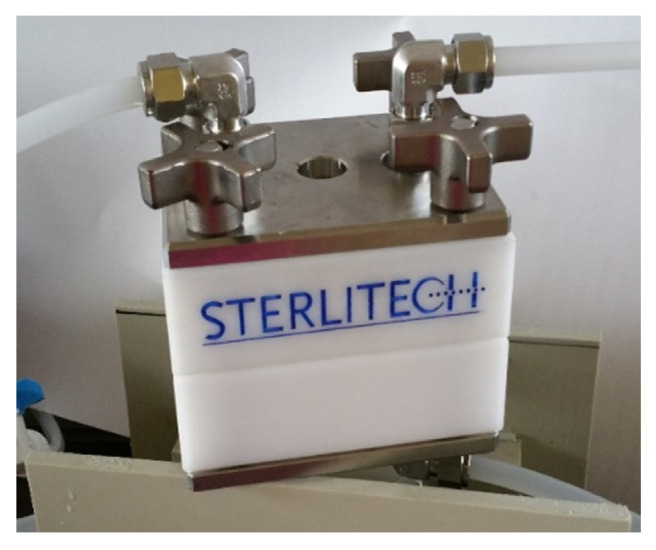
CF042D-FO Sterlitech laboratory module.

As flow conditions affected mass transfer conditions, further FO experiments were conducted to investigate the effect of linear flow velocity on fluxes across the membrane. These were conducted in the single pass CF042D-FO Sterlitech laboratory module (Figure 1) equipped with a flat-sheet Aquaporin membrane with an effective surface of 42 cm^2^ (active membrane length 9.2 cm), and a 0.7 mm thick spacer. The following draw solutions were used in experiments:

- 40% glycerol, 3% NaCl;
- 40% glycerol, 5% NaCl;
- 40% glycerol, 3% NaCl, 5% methanol;
- 40% glycerol, 3% NaCl, 10% methanol;
- 60% glycerol, 5% methanol;
- 70% glycerol, 3% NaCl, 8% methanol;
- 80% glycerol, 3% NaCl;
- 80% glycerol, 5% NaCl;
- 80% glycerol, 3% NaCl, 5% methanol;
- 80% glycerol, 3% NaCl, 10% methanol.

Experiments in co- and counter-current flow mode, using the same linear flow velocity on both sides of the membrane, were conducted to determine the effect of flow mode on the water flux.

Fluxes of water, salt, and glycerol were determined based on volume change, density, and measured compositions (analyzed by ion and gas chromatography) of the obtained solutions. Aliquots (0.5 mL, for RO and NF every 60 min, for FO every 15 min) were collected during these experiments. In all experiments, the membranes faced draw solutions with an active layer and DI water at the support layer side; the main role of the membrane in FO was to allow water transport and hinder transport of any other compounds.

## 3. Results

### 3.1. Osmotic Pressure of Glycerol Solutions upon Composition

**Figure 2:**
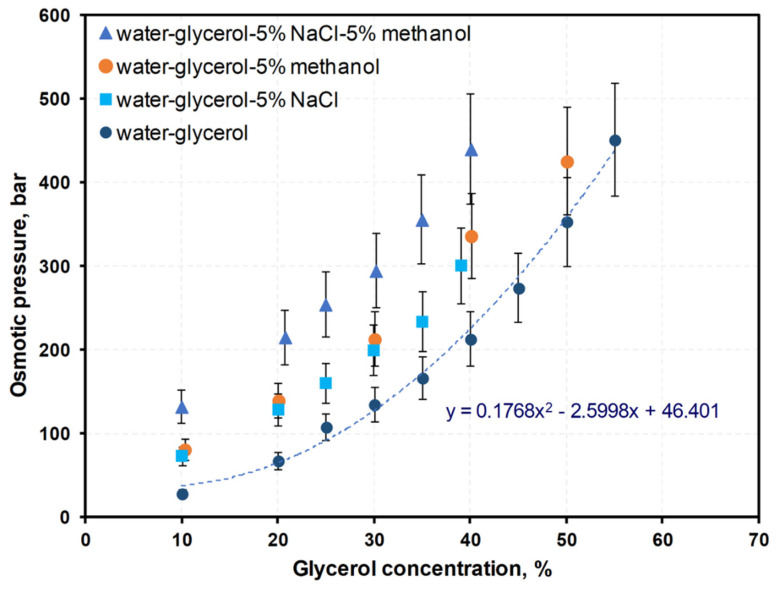
The effect of salt and methanol on osmotic pressure, calculated based on freezing point depression measurements in water–glycerol–NaCl–methanol systems.

The osmotic pressures of the solutions were calculated based on freezing point depression measurements in water–glycerol, water–glycerol–salt, water–glycerol–salt–methanol systems. Raw measurement data are shown in Supplementary Materials. Figure 2 shows the effect of the addition of common waste glycerol impurities on osmotic pressure.

Two empirical models describing the osmotic pressure dependency upon composition for systems in this study were determined by multiple regression using Statistica 13.1 software.

For water–glycerol solutions (Figure 2):

π = 0.1768 × C_g_^2^ − 2.5998 × C_g_ + 46.401;

R = 0.99685142; R^2^ = 0.99371276; *p* < 0.00005; S_e_ = 12.670.

The fitting curve is shown on Figure 2.

For water–glycerol–salt and water–glycerol–salt–methanol solutions (Supplementary Materials Figures S1–S3, Figure 2):

π = –1521.15 + 2964.42 × Wg + 3867.96 × Wmet + 1548.12 × W_H_2_O_^2^ + 3629.31 × W_NaCl_;

R = 0.99561; R2 = 0.99123619; *p* < 0.00005; S_e_ = 10.289,

where π = osmotic pressure, bar; W = mass fraction; C = percentage concentration, %; subscripts g, H_2_O, NaCl denote glycerol, water, and sodium chloride, respectively; S_e_ = standard error of estimate.

Extrapolation of the above equations was used to determine the osmotic pressure of glycerol in solutions when concentrations exceeded the measurement range.

### 3.2. Selection of Membranes for FO of Glycerol Solutions

**Figure 3:**
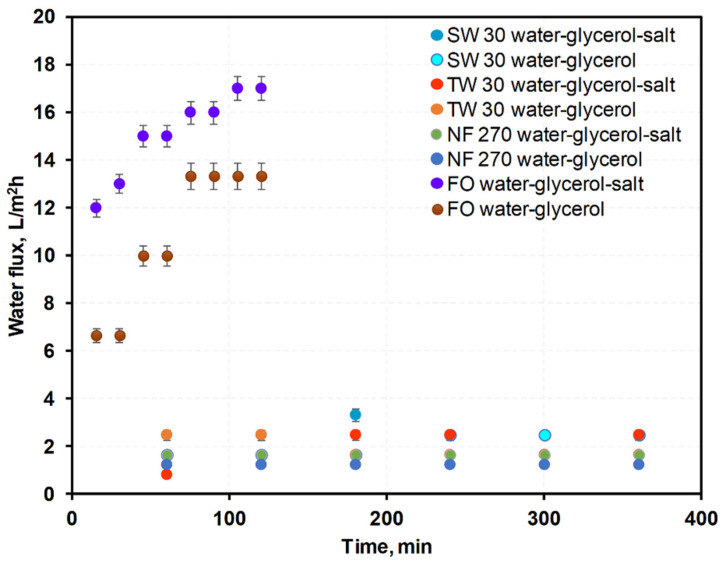
Measured water fluxes through membranes for water–glycerol and water–40% glycerol–3% NaCl solutions.

**Figure 4:**
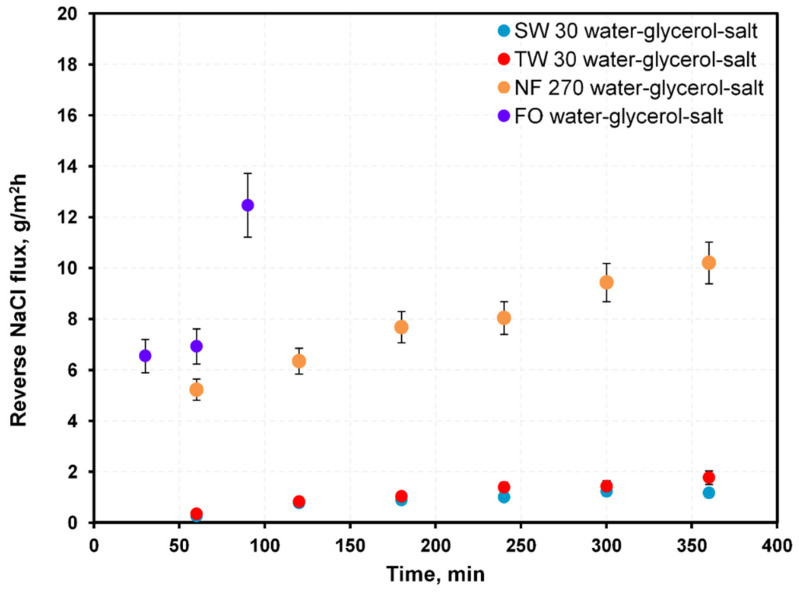
Measured reverse NaCl fluxes through membranes for water–40% glycerol–3% NaCl solutions.

FO membranes should allow high water flux, low reverse flux, and show high durability under a high osmotic pressure difference. Historically, RO or NF membranes were used in forward osmosis due to a lack of dedicated FO membranes. Recently, membranes dedicated to FO became available. These FO membranes have thinner support layers, which enhance their performances by up to 50% [27]. Currently, FO membranes with two active layers on both support sides were investigated, which were designed to counteract scaling and fouling [16]. These may be particularly useful when using waste glycerol as a draw solution as they are intended for use with draw solutions of high viscosity. In this work, performance of three membranes typically used in pressure-driven membrane processes (RO:SW30, TW30; NF:NF270) were compared with an FO Aquaporin membrane with respect to water, glycerol, salt, and methanol fluxes. The measured diffusion fluxes of water and NaCl through each membrane are shown in Figure 3 and Figure 4.

Among the membranes, FO membranes showed the highest water fluxes, and the RO membranes showed the lowest, though for the NaCl flux, the opposite was the case. The NF membranes showed water fluxes higher than RO membranes, and NaCl flux lower than the FO membranes (Figure 3 and Figure 4). High water fluxes across the FO membranes were credited to their porous nature, hydrophilic properties, and small thicknesses. RO and NF processes are normally driven by hydraulic pressure differences, and, according to the widely used solution–diffusion transport model, water flux through a membrane is proportional to the sum of the hydraulic and osmotic pressure differences. In so-called pressure-assisted FO, additional hydraulic pressure is applied to improve water flux. However, the main reason for testing RO and NF membranes was to hinder reverse fluxes of impurities. RO and NF membranes have low bulk porosity, no surface pores, a hydrophobic support layer, and thick dense support that results in a lower water flux than FO membranes. The RO and NF membranes had high mass transport resistance, which led to severe concentration polarization that decreased the water flux.

**Figure 5:**
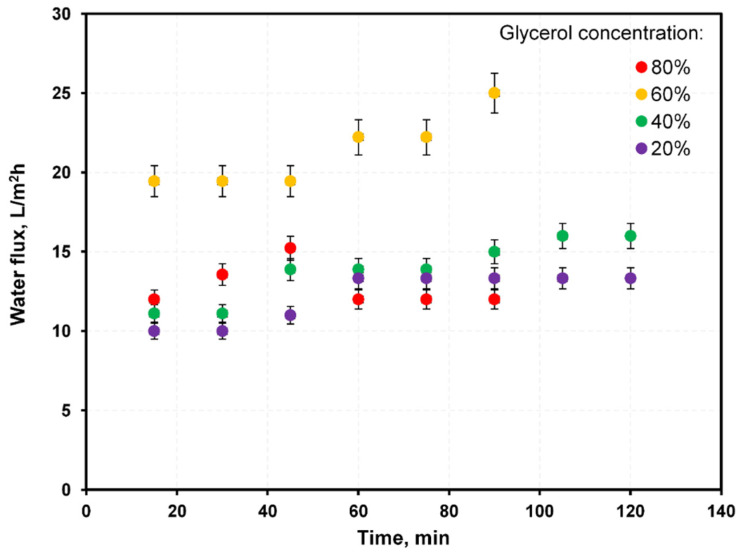
The measured water flux through FO Aquaporin membrane under different glycerol concentrations in a water–glycerol system.

**Figure 6:**
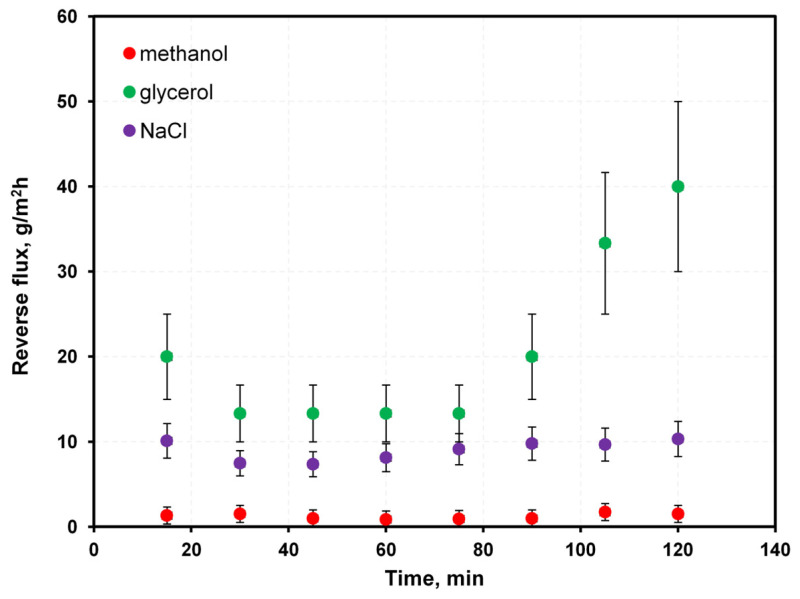
The measured reverse fluxes of impurities through FO Aquaporin membrane in a water–40% glycerol–3% salt–5% methanol system.

A literature overview regarding different draw solution performances applied to FO using DI as the feed shows that typical water flux was within 5–40 L/m^2^h. Under the same conditions, reverse fluxes of substances contained in draw solutions were generally <100 g/m^2^h but exceeded 500 g/m^2^h in some cases [27,28,29,30,31,32,33]. The measured water flux through the Aquaporin membrane (Figure 5) was 10–25 L/m^2^h. Generally, water flux increased with glycerol concentration in the draw solution, except for the 80% glycerol solution, caused by the increase of external concentration polarization in FO. The 80% glycerol solution had a significantly higher density (1.21 g/cm^3^) and viscosity (60.1 mPa·s) than the other solutions. The 80% glycerol solution showed a water flux significantly lower than the 70% glycerol (viscosity 22 mPa·s) solution and similar to 20–40% glycerol solutions (densities ~1.05 g/cm^3^, viscosities between 1.7–3.8 mPa·s). The reverse flux (Figure 6) of methanol was <0.2 g/m^2^h, which was rather low. As expected, a high concentration of glycerol in the draw solution resulted in a high reverse flux of glycerol to the feed (10–50 g/m^2^h). For NaCl, the reverse flux was ~10 g/m^2^h—relatively high, considering that the salt concentration in the draw solution was much lower than that of glycerol.

Among the tested membranes, Aquaporin FO was the most suitable for FO due to its high water flux and comparatively low reverse flux. This membrane was used for additional testing in a single-pass module.

**Figure 7:**
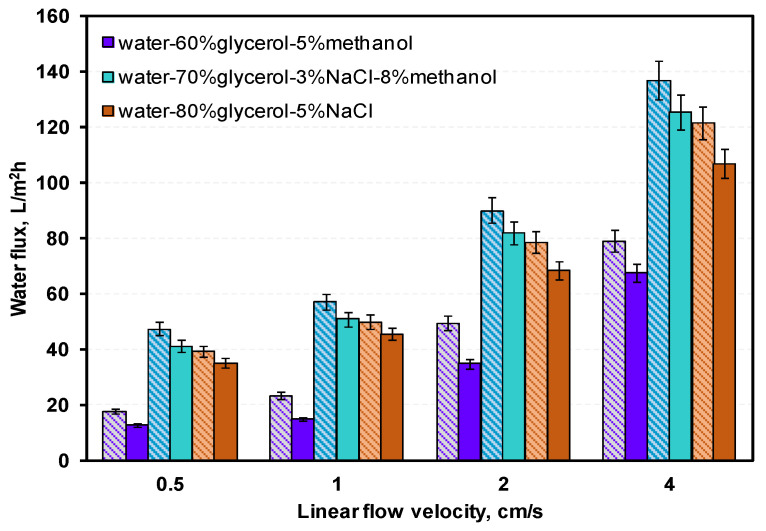
Water flux dependence of linear flow velocity in co-current (solid bars) and counter-current (striped bars) flow mode for different water–glycerol–salt–methanol draw solutions.

Flow mode and velocity affect hydrodynamics in boundary layers adjacent to the membrane and thus the FO effective driving force; therefore the water flux, which is the main parameter of FO processes and should be maximized. The effects of co-current or counter-current flow on water flux was investigated; the same linear flow velocity was applied to both draw and feed solutions. The observed dependence of water flux on linear flow velocity measurements for both flow modes (Figure 7) indicated that in counter-current water flux was greater in all cases. Thus, further experiments with various feed compositions were conducted in counter-current flow mode.

**Figure 8:**
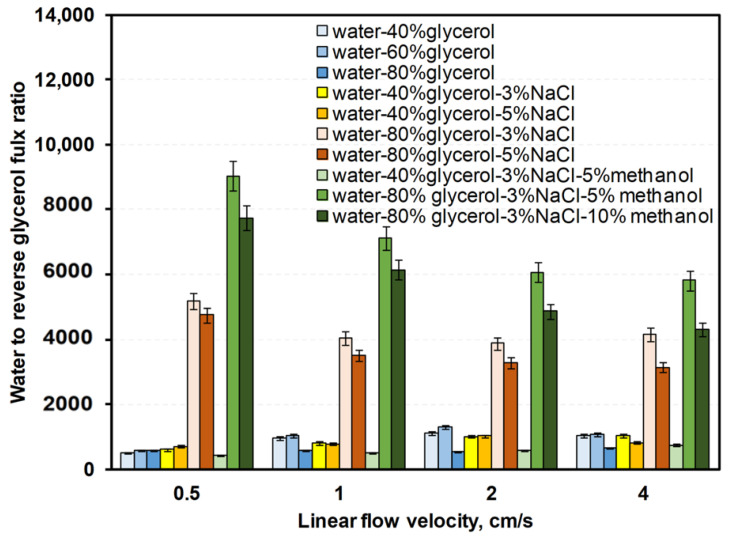
Water-to-reverse glycerol mass flux ratios for various linear flow velocities in counter-current FO with different water–glycerol–salt–methanol draw solutions.

**Figure 9:**
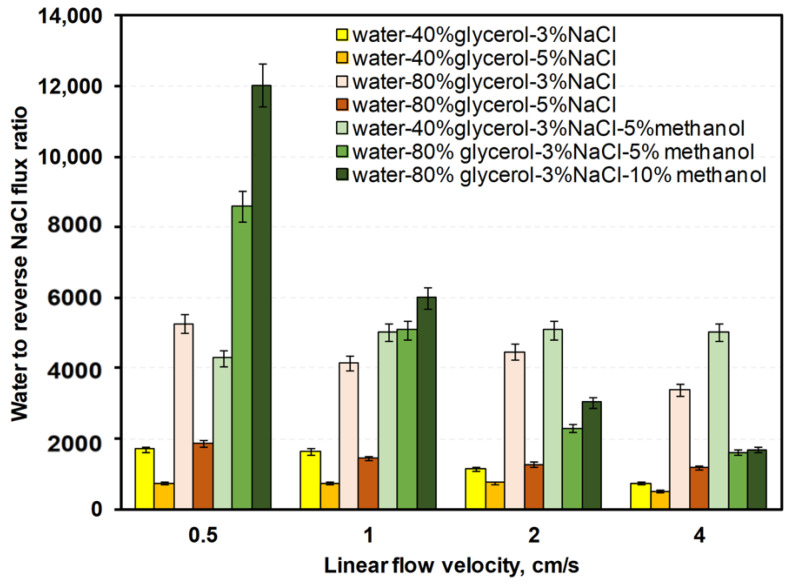
Water-to-reverse NaCl mass flux ratios for various linear flow velocities in counter-current FO with different water–glycerol–salt–methanol draw solutions.

**Figure 10:**
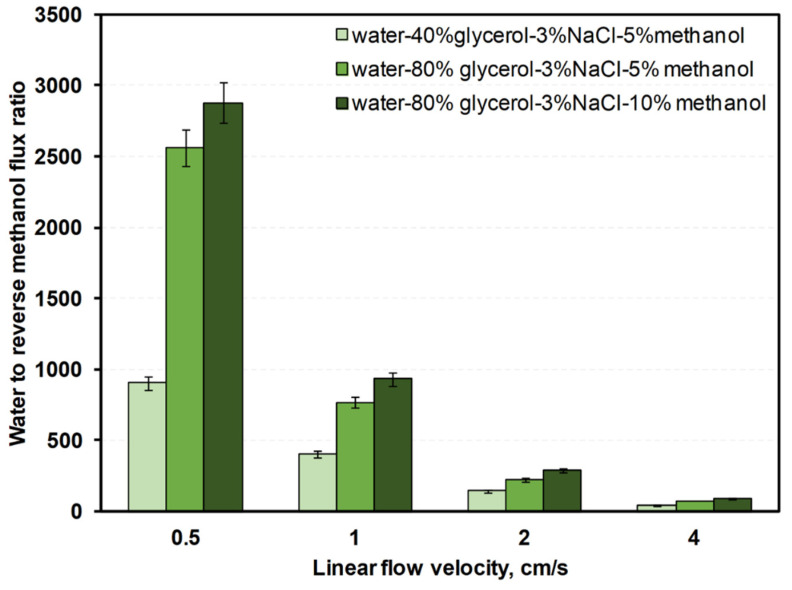
Water-to-reverse methanol mass flux ratios for various linear flow velocities in counter-current FO with different water–glycerol–salt–methanol draw solutions.

The Supplementary Materials contain data on water, NaCl, glycerol, and methanol fluxes (Figures S4–S7). Figure 8, Figure 9 and Figure 10 show ratios of water flux to reverse fluxes of impurities for drawing solutions under linear flow velocities of 0.5–4 cm/s. Water flux increased with linear flow velocity and with draw solution concentration (Figure 7). Given that both factors increase the driving force, a higher linear flow velocity improves mass transfer in the boundary layer and the draw solution concentration increases the osmotic pressure difference. Reverse flux of glycerol varied less with linear flow velocity than reverse flux of NaCl or methanol (Figure 8, Figure 9 and Figure 10). However, at a linear flow velocity of 4 cm/s, the methanol reverse flux was very high (>1400 g/m^2^h), which excludes it from practical application. This may be due to the similarities of methanol and water and its higher permeability through the membrane than NaCl or glycerol. NaCl reverse flux is within the usual experimentally obtained range (typically 0–80 g/m^2^h) [34].

For a linear flow velocity of 2 cm/s, the water flux exceeded the typical FO water flux (5–40 L/m^2^h), and reverse fluxes of salt and glycerol in most cases were lower than typical FO processes (100 g/m^2^h). The methanol reverse flux was approximately five times lower than the linear flow velocity (4 cm/s). Taking the above into account, the 2 cm/s linear flow velocity was the most suitable for FO in this study.

## 4. Discussion

This study shows the potential application of crude glycerol as a draw solution for FO. The osmotic pressure difference between the draw and feed solutions is the main driving force for FO and determines its efficiency. The dependencies of osmotic pressure upon concentration and composition in glycerol–water, glycerol–NaCl–water, and glycerol–NaCl–methanol–water systems determined in this paper allow for better draw solution selection, both in terms of providing high driving force and determining the process endpoint.

**Figure 11:**
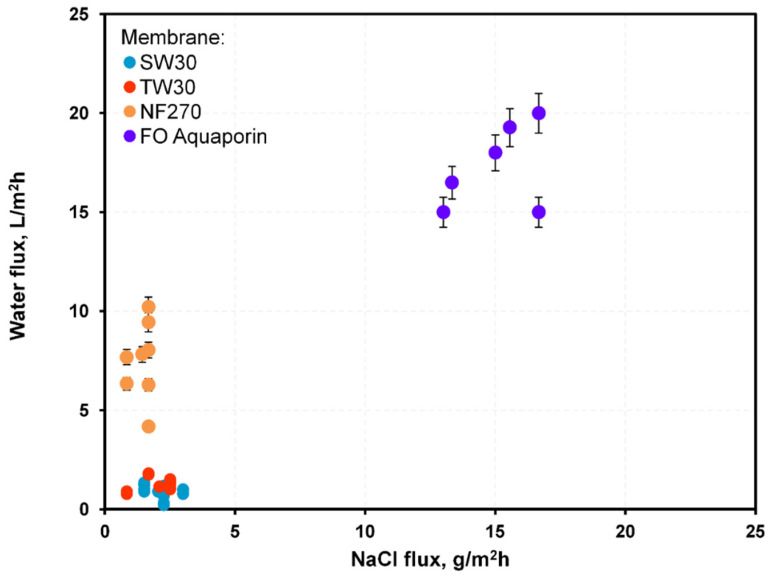
Ratio of measured water to salt fluxes in a water–40%glycerol–3% NaCl system through the membranes in this study.

Another important aspect of FO is proper membrane selection. High water flux and minimal reverse flux are crucial. High reverse flux of impurities causes feed contamination, which is unwanted when utilizing FO as a dehydration method. Dense membranes, like those for RO or NF, mitigate salt transport and were tested as potentially favorable for FO despite their significant internal concentration polarization. Figure 11 shows the ratio of water to salt flux measured in diffusion experiments through the membranes in this study.

The average ratio of water to salt (mass) flux during diffusion from the water–40% glycerol–3% NaCl system was ~3100 for SW30, 1600 for TW30, 880 for FO Aquaporin, and 200 for NF 270 membranes. Although experimental data showed the ratio of water flux to NaCl through both RO exceeded the FO Aquaporin membrane, a water flux through these membranes almost 10 times lower was unfavorable for the FO process. Nevertheless, the membrane dedicated to FO showed the highest water flux (almost 10 times higher than RO membranes), which corresponded to a shorter process and smaller installed membrane area (lower capital cost) than membranes for other pressure-driven membrane processes (NF, RO).

The effect of flow on FO performance was analyzed. Based on single pass experimental results, a 2 cm/s linear flow velocity was most favorable, ensuring both high water flux and low reverse impurity flux. Additionally, the counter-current water flux was 8–15% higher than the co-current; thus counter-current flow mode was more favorable for FO. Under these conditions, depending on the composition of the draw solution, fluxes of water, NaCl, glycerol, and methanol were 30–100 L/m^2^h, 10–70 g/m^2^h, 15–100 g/m^2^h, and 300–410 g/m^2^h, respectively. The higher than typical (10–40 L/m^2^h) water flux might be due to high concentrations (thus osmotic pressures) of the tested draw solutions—in this study, this was 200–1200 bar. Typically, salt solutions range from 1–4 M, which corresponds to 20–600 bar [35]. Despite the high viscosities and densities, the very high osmotic pressure of waste glycerol solution compensates for the occurrence of external concentration polarization (CP), and still enables a significant water flux.

These results show that using waste glycerol as a draw solution in FO might assist the purification of waste glycerol from biofuel production. In this process, the purpose of FO was not to recover pure water but to facilitate selective transport of water from one process solution to another. While obtaining pharmaceutical grade glycerol, 80% crude glycerol is diluted from 40–50% to decrease density and viscosity, which are problematic for desalination methods. The diluted glycerol solution is then purified, and organic pollutants and salts are removed. The purified 40% glycerol solution is then concentrated to >90% by vapor compression (VC), which has high energy use. Thanks to FO, crude glycerol dilution and purified glycerol concentration can occur simultaneously. Methanol present in the waste glycerol might be problematic due to its diffusion to the feed solution and could be removed either before or after FO by ion exchange.

These results indicate the feasibility of using waste glycerol as a draw solution in FO. However, in commercial use of waste glycerol as a draw solution, the application of additional technology (e.g., ion exchange) might be required to meet high-purity standards. The application of feed solutions other than DI will decrease the reverse flux of NaCl, methanol, and glycerol as the osmotic pressure difference—the FO driving force—and diffusion transport decrease. Application of crude glycerol as the draw solution for dewatering in bio-processes, to purify crude glycerol, and in other industries, may mitigate the problems of utilizing significant amounts of this by-product.

## 5. Conclusions

Waste glycerol is a feasible draw solution due to its high osmotic pressure, which ensures a high driving force for FO. However, pure glycerol might not be an ideal draw solution due to its high density and viscosity, though in some cases it might be applied in forward osmosis. As expected, osmotic pressure increased with glycerol concentration—and exceeded 600 bar for 80% glycerol, making it one of highest among substances used as draw solutions in FO. The impurities present in waste glycerol (composed of 20–80% glycerol, 5% methanol, 1–5% NaCl) increase its osmotic pressure; however, they (especially methanol) tend to transport through the membrane to the feed solution (reverse flux), which is a serious drawback, as it contaminates the feed solution during FO. Use of other dense membranes (designed for RO or NF) was tested to limit this; however, the water flux was unsatisfactory and therefore they were not applicable. The concentrations/dilutions of glycerol solutions with FO were optimized with flow condition selection to minimize concentration polarization: a counter-current flow mode and a sufficiently high linear flow velocity.

Noticeably, a reverse flux of impurities did not exclude using waste glycerol as a draw solution in FO. Methanol and salts were removed before FO to prevent its transport to feed solutions or were removed from feed solutions in other FO processes.

Our results should serve as a guide in the design of FO processes using waste glycerol.

## 6. Patents

PL 236277 B1 Sposób zatężania oczyszczonych roztworów gliceryny.

## Figures and Tables

**Table 1:** Characteristics of the membranes tested.

Type of Membrane	Membrane Purpose ****	Pore Size/MWCO	Rejection	Polymer	pH Range	Applied Pressure	Flux
SW30 * (Dow Filmtec)	RO	~100 Da	99.6% NaCl	Polyamide TFC	2–11	55 bar	30.6–40.8 l/m^2^h;
TW30 ** (Dow Filmtec)	RO	~100 Da	99.5% NaCl	Polyamide TFC	2–11	15 bar	51.0–54.4 l/m^2^h;
NF270 *** (Dow Filmtec)	NF	~200–400 Da	99.2% MgSO_4_	Polyamide TFC	2–11	9 bar	51.3–52l/m^2^h;
Aquaporin inside forward osmosis membrane (Aquaporin)	FO	No data	NaCl reverse flux <2.5 g/m^2^/hr (H_2_O vs. 1 M NaCl; FO mode)	Protein-embeddedpolyamide AquaporinTFC	2–11		>7 (H_2_O vs. 1 M NaCl; FO mode)

Process parameters determined by manufacturers in the following test conditions: * 32,000 ppm NaCl feed stream (5 ppm boron, SW30HRLE), 25 °C and 2–8% recovery; ** 2000 ppm NaCl feed stream XLE & LP, 500 ppm NaCl feed stream, 25 °C and 5–15% recovery; *** 2000 ppm MgSO_4_, and 25 °C and 15% recovery. **** According to the manufacturer.

## Data Availability

The data presented in this study are available in supplementary material.

## Supplementary Materials

The following supporting information can be downloaded at: https://www.mdpi.com/article/10.3390/membranes12010044/s1, Figure S1: The effect of salt and glycerol concentration on osmotic pressure, calculated based on freezing point measurements, for water–glycerol–NaCl systems.; Figure S2: The effect of salt and glycerol concentration on osmotic pressure, calculated based on freezing point measurements, for water–glycerol–NaCl–5% methanol solutions.; Figure S3: The effect of salt and glycerol concentration on osmotic pressure, calculated based on freezing point measurements, for water–glycerol–NaCl–10% methanol solutions.; Figure S4: Measured water fluxes under various linear flow velocities during FO experiments in water–glycerol–NaCl–methanol systems.; Figure S5: Measured reverse glycerol fluxes under various linear flow velocities during FO experiments in water–glycerol–NaCl–methanol systems.; Figure S6: Measured reverse NaCl fluxes under various linear flow velocities during FO experiments in wa-ter–glycerol–NaCl–methanol systems.; Figure S7: Measured reverse methanol fluxes under various linear flow velocities during FO experiments in water–glycerol–NaCl–methanol systems.
