# Supplementary material for: Application of Waste Glycerol as a Draw Solution for Forward Osmosis
Source: Membranes (Basel). 2021 Dec 29;12(1):44. doi: 10.3390/membranes12010044 (PMC8780772; doi:10.3390/membranes12010044)
Supplement: Supplementary file 1 [file membranes-12-00044-s001.zip › membranes-1468421-supplementary.pdf]

membranes

Article

Application of Waste Glycerol as a Draw Solution for Forward Osmosis

Ewa Bernacka [1,*], Hanna Jaroszek [1], Marian Turek [2], Piotr Dydo [2], Dymitr Czechowicz [2] and Krzysztof Mitko [2]

[1] PolymemTech Sp. z o.o., Ul. Wołodyjowskiego 46, 02-724 Warsaw, Poland; h.jaroszek@gmail.com
[2] Faculty of Chemistry, Silesian University of Technology, Ul. B. Krzywoustego 6, 44-100 Gliwice, Poland; marian.turek@polsl.pl (M.T.); piotr.dydo@polsl.pl (P.D.); dymitr.czechowicz@polsl.pl (D.C.); krzysztof.mitko@polsl.pl (K.M.)
* Correspondence: laskowska.ewi@gmail.com

Citation: Bernacka, E.; Jaroszek, H.; Turek, M.; Dydo, P.; Czechowicz, D.; Mitko, K. Application of Waste Glycerol as a Draw Solution for Forward Osmosis. *Membranes* **2022**, *12*, 44. https://doi.org/10.3390/membranes12010044

Academic Editors: Boguslaw Kruczek

Received: 2 November 2021
Accepted: 21 November 2021
Published: 29 November 2021

Publisher's Note: MDPI stays neutral with regard to jurisdictional claims in published maps and institutional affiliations.

Copyright: © 2021 by the authors. Licensee MDPI, Basel, Switzerland. This article is an open access article distributed under the terms and conditions of the Creative Commons Attribution (CC BY) license (https://creativecommons.org/licenses/by/4.0/).

Supplementary material

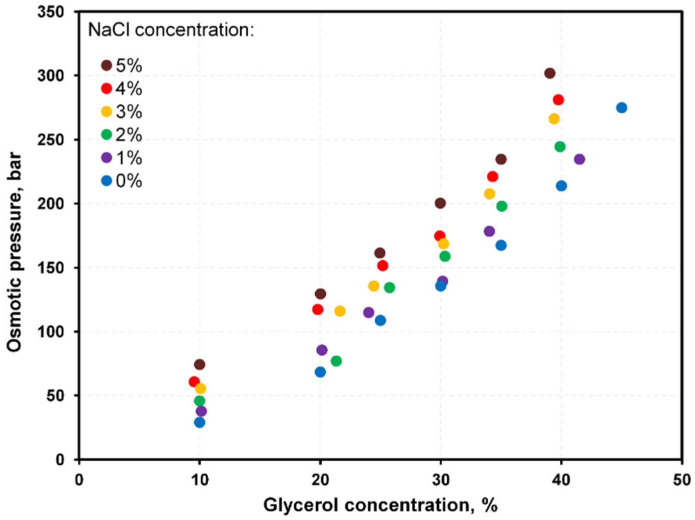

Figure S1. The effect of salt and glycerol concentration on osmotic pressure, calculated based on freezing point measurements, for water–glycerol–NaCl systems.

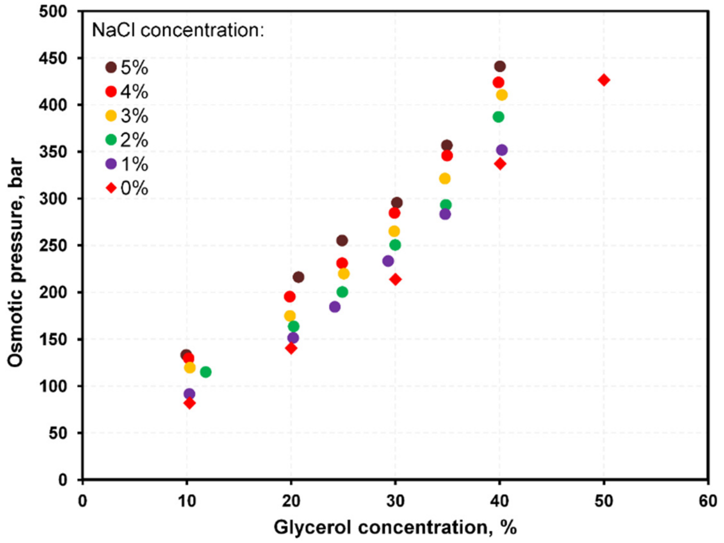

Figure S2. The effect of salt and glycerol concentration on osmotic pressure, calculated based on freezing point measurements, for water–glycerol–NaCl–5% methanol solutions.

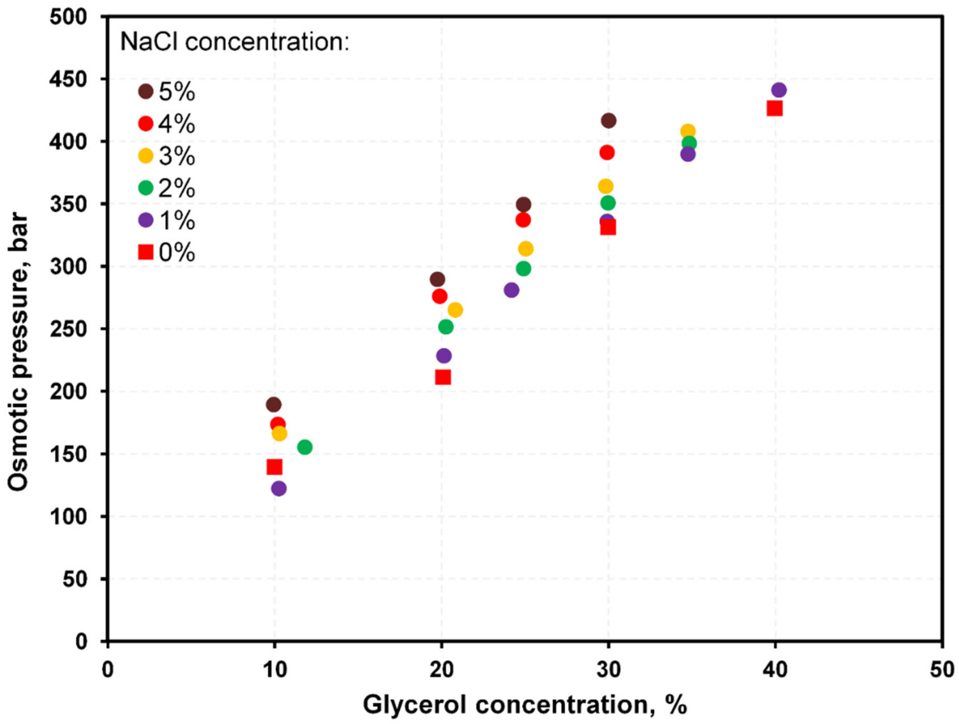

Figure S3. The effect of salt and glycerol concentration on osmotic pressure, calculated based on freezing point measurements, for water–glycerol–NaCl–10% methanol solutions.

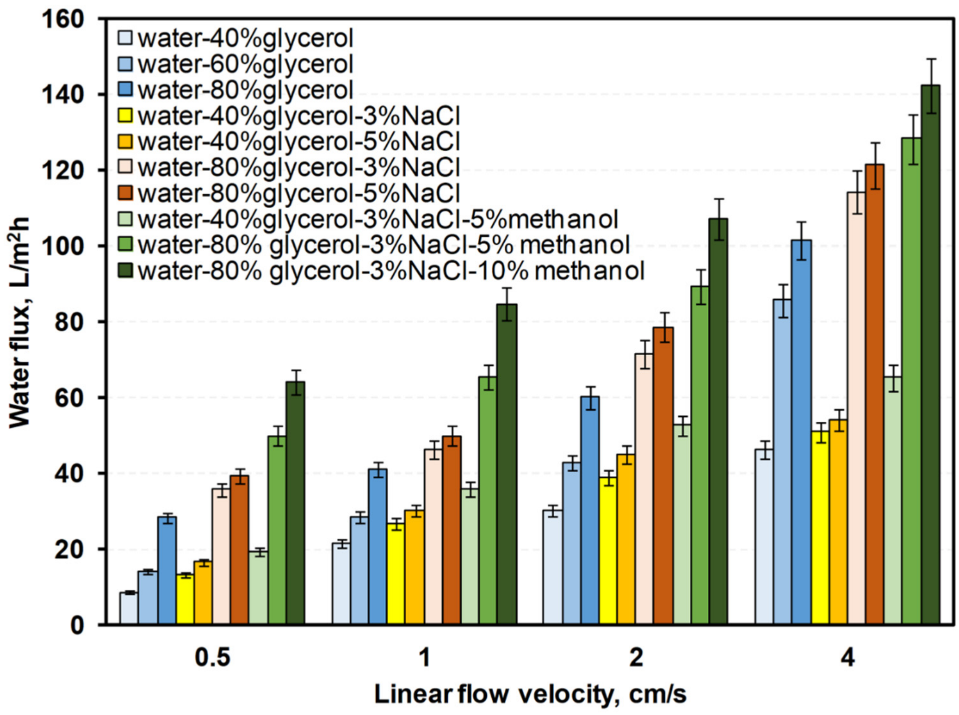

Figure S4. Measured water fluxes under various linear flow velocities during FO experiments in water–glycerol–NaCl–methanol systems.

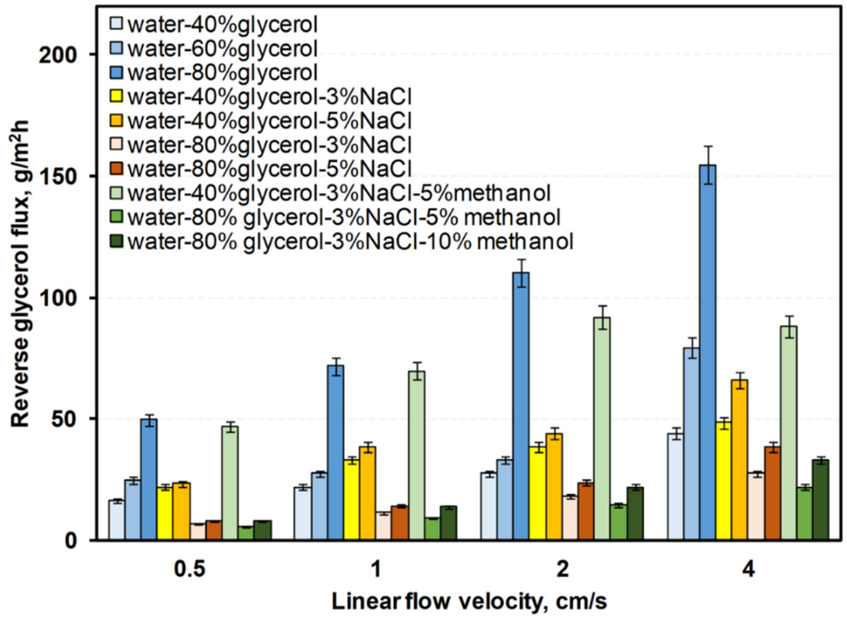

Figure S5. Measured reverse glycerol fluxes under various linear flow velocities during FO experiments in water–glycerol–NaCl–methanol systems.

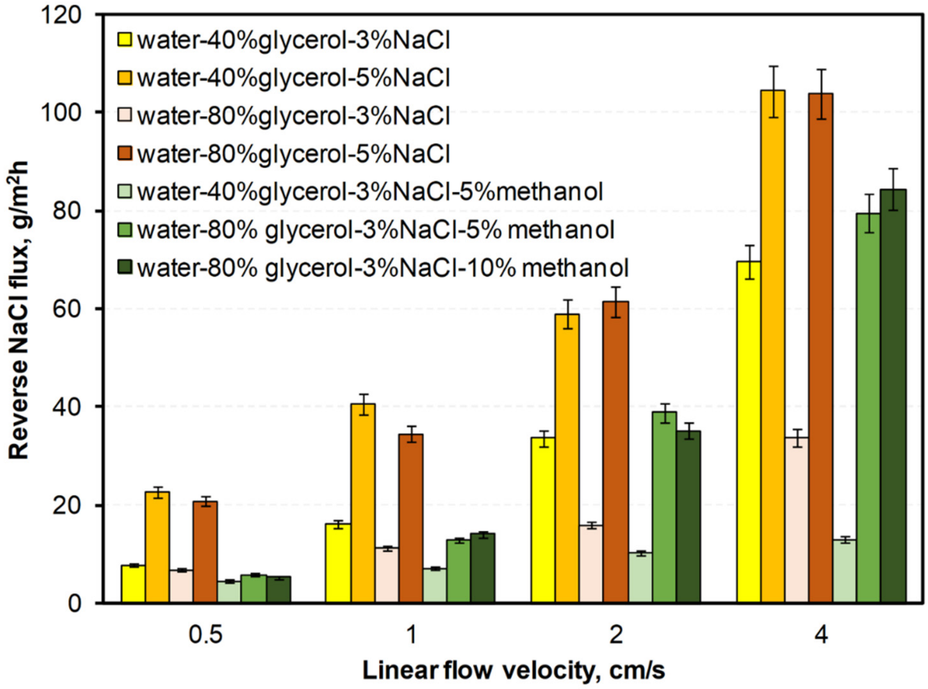

Figure S6. Measured reverse NaCl fluxes under various linear flow velocities during FO experiments in water–glycerol–NaCl–methanol systems.

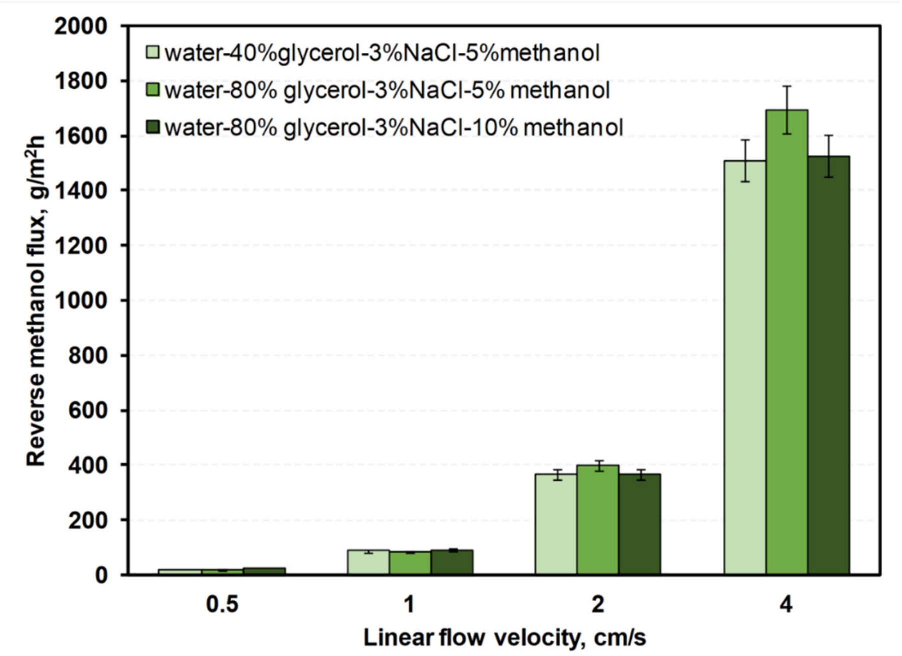

Figure S7. Measured reverse methanol fluxes under various linear flow velocities during FO experiments in water–glycerol–NaCl–methanol systems.
